# Factors associated with sarcopenia and exploratory thresholds for low muscle strength in people living with the human immunodeficiency virus

**DOI:** 10.1186/s12879-026-12961-z

**Published:** 2026-03-24

**Authors:** Jaine Alves Ximenez, Maria Claudia Bernardes Spexoto

**Affiliations:** Faculdade de Ciências da Saúde, Universidade Federal da Grande Dourados, Programa de Pós-Graduação em Alimentos, Nutrição e Saúde, Rodovia Dourados/Itahum, Km 12 – Unidade II, Dourados, 364 MS Brazil

**Keywords:** HIV, Sarcopenia, Handgrip strength, Quality of life, Screening

## Abstract

**Background:**

The revised European Working Group on Sarcopenia in Older People (EWGSOP2) criteria are widely used among people living with HIV (PLHIV). However, accumulating evidence suggests that conventional thresholds may underestimate sarcopenia in PLHIV and fail to capture early functional decline. This study explored handgrip strength (HGS) values ​​associated with poorer quality of life (QoL) to assist early functional screening, and identified factors associated with sarcopenia.

**Methods:**

Cross-sectional study in PLHIV on antiretroviral therapy. We collected sociodemographic, clinical, lifestyle and QoL variables; defined sarcopenia using EWGSOP2 algorithm; and evaluated HGS accuracy for discriminating poorer QoL (WHOQOL-HIV Bref median). We present exploratory HGS thresholds and factors associated with sarcopenia in adjusted models.

**Results:**

A total of 105 PLHIV participated (mean age 44.6 ± 13.5 years; 51.4% men). The HGS thresholds with the best accuracy for poorer QoL were ≤ 33 kg for men (sensitivity: 72.0%, specificity: 65.52%, Youden’s index: 0.37, AUC: 0.66) and ≤ 26 kg for women (sensitivity: 88.0%, specificity: 53.85%, Youden’s index: 0.41, AUC: 0.70). CD4 + T-cell count ≤ 200 cells/mm³ were associated with sarcopenia in both constructs (adjusted OR ≈ 4). Poorer QoL was associated with sarcopenia when using HGS ≤ 33/26 kg thresholds (OR = 5.12; 95% CI: 2.02–12.98).

**Conclusion:**

Sarcopenia was associated with immunosuppression in both models. HGS thresholds of ≤ 33/26 kg may be useful for early outpatient screening of functional vulnerability in PLHIV, while conventional thresholds (< 27/16 kg) may better reflect more severe clinical settings. Findings should be interpreted as exploratory and considered alongside clinical judgment.

**Clinical trial number:**

Not applicable.

**Supplementary Information:**

The online version contains supplementary material available at 10.1186/s12879-026-12961-z.

## Introduction

Sarcopenia is a significant muscular disease among people living with HIV (PLHIV), with a multifactorial etiology [1, 2], and its reported prevalence in the literature varies depending on the diagnostic criteria adopted. A meta-analysis by Oliveira et al. [3], including 2,267 participants from 13 studies, estimated a global prevalence of 24.1% (95% CI: 17.8–31.0) and indicated that PLHIV have a 6.1-fold higher risk of developing sarcopenia compared to individuals without the human immunodeficiency virus (HIV) (OR: 6.1; 95% CI: 1.1–33.5). Similarly, SeyedAlinaghi et al. [2], in a systematic review including 2,592 PLHIV aged 30 to 60 years, reported prevalence rates ranging from 10% to 24%, reinforcing the need to standardize sarcopenia assessment criteria in this population.

Sociodemographic and clinical factors related to HIV infection have been associated with sarcopenia among PLHIV, including older age, male sex, low educational attainment, unemployment, smoking, excessive alcohol consumption, longer duration of HIV infection, lower CD4 + T-cell count, hospitalization, longer exposure to antiretroviral therapy (ART), multimorbidity, and polypharmacy [4–6]. However, few studies have investigated the prevalence of sarcopenia and its associated factors among PLHIV in Brazil [1, 4, 5, 7–9]. Another relevant factor to consider is quality of life (QoL) and its domains, which are often analyzed as outcomes in studies on HIV [10, 11] but may also serve as an exposure variable, influencing the development of sarcopenia in this population.

In Brazil, most studies on sarcopenia in PLHIV adopt the revised algorithm and criteria proposed by the European Working Group on Sarcopenia in Older People (EWGSOP2) [4, 5, 8, 12, 13], which consider reduced muscle strength as the primary criterion, followed by the assessment of muscle mass and physical performance. According to this recommendation, the established cutoff points for defining low muscle strength are < 27 kg for men and < 16 kg for women. However, applying these cutoff points may not adequately reflect the reality of this population, potentially leading to an underestimation of the true prevalence of sarcopenia.

Previous studies have highlighted limitations in applying existing sarcopenia assessment criteria to people living with HIV, underscoring the need for approaches that better capture clinically relevant outcomes in this population [2, 8, 14]. Martins et al. [8], in a Brazilian study involving 218 PLHIV, used the EWGSOP2 diagnostic criteria and found a sarcopenia prevalence of 8.7%, which the authors considered low. They suggested that using these criteria may have underestimated the prevalence of sarcopenia, given that PLHIV are generally younger, and that applying criteria specific to PLHIV could have enabled earlier identification of functional impairment related to sarcopenia in this population [8].

However, to date, no study has compared cutoff points or proposed specific cutoff points for determining low muscle strength in PLHIV that have been tested against health outcomes, even when determined in cross-sectional studies. In this context, it is important to emphasize the need to develop specific diagnostic criteria for sarcopenia in PLHIV, prioritizing definitions capable of predicting unfavorable outcomes such as poorer quality of life [14]. Therefore, our objectives were to (i) present exploratory handgrip strength (HGS) thresholds associated with poorer QoL to assist early functional screening; and (ii) identify factors associated with sarcopenia.

## Methods

### Study population

PLHIV with confirmed diagnoses recorded in medical charts, treated at both outpatient or inpatient levels, and currently receiving ART, aged ≥ 20 years, of both sexes, whether literate or illiterate, were included as long as they had sufficient cognitive and physical capacity to complete the study tests. Individuals with a total inability to ambulate without assistance, cognitive impairment, and severe psychiatric disorders recorded in medical charts, edema or restrictions preventing handgrip strength assessment, and patients diagnosed during their first hospitalization were not included. Although sarcopenia is classically age-related, PLHIV may experience earlier declines in muscle strength due to chronic inflammation, comorbidities, and antiretroviral therapy effects. We therefore included adults aged ≥ 20 years to capture potential early onset in this population.

The study was conducted at the University Hospital of the Federal University of Grande Dourados (HU-UFGD) and the Specialized Care Service/Testing and Counseling Center (SAE/CTA), both located in Dourados, Mato Grosso do Sul, Brazil, between April 2023 and December 2024. HU-UFGD is a referral center for public healthcare in 33 municipalities across the Grande Dourados region and consists of a general hospital providing medium- and high-complexity care. It has 34 inpatient medical clinic beds, 12 of which are dedicated to infectious diseases, and 25 outpatient consultation rooms, two of which are exclusively for infectious diseases [15]. SAE/CTA serves as a referral center for individuals with sexually transmitted infections (STIs) and people living with HIV, offering testing, diagnosis and treatment, pre- and post-exposure prophylaxis, immunization, and condom distribution.

Participant identification was carried out using appointment schedules, daily nursing censuses, the hospital’s electronic system, and information provided by the multidisciplinary teams at both study sites. Weekly, a trained researcher screened patients who met the eligibility criteria. Data collection was conducted in a manner that ensured confidentiality and privacy, safeguarding patients’ personal information.

### Risk of sarcopenia

The risk of sarcopenia was assessed using the SARC-F [16] and SARC-Calf [17] questionnaires, both validated in Portuguese. The SARC-F evaluates five components: strength, assistance in walking, rising from a chair, climbing stairs, and falls; each scored from 0 to 2 points, where 0 indicates better functional ability, and 2 indicates worse functional ability. A total score ≥ 4 (maximum of 10 points) was considered indicative of sarcopenia risk.

The SARC-CalF includes the same five items as the SARC-F, along with calf circumference (CC). CC scored 10 points if the measurement was ≤ 34 cm for men or ≤ 33 cm for women, and 0 points if the measurement exceeded these cutoff points. Calf circumference was measured using a flexible, non-stretchable tape measure, following Lohman’s criteria [18]. The maximum possible score on the SARC-CalF is 20 points; a total score ≥ 11 indicates risk of sarcopenia, while a score < 11 suggests absence of sarcopenia signs.

It is important to note that the SARC-F and SARC-CalF were not used as screening tools for sarcopenia in this study, as the evaluation was based directly on reduced muscle strength.

### Muscle strength

Muscle strength was assessed through handgrip strength (HGS, kg) using a SAEHAN^®^ hydraulic hand dynamometer, model SH5001 (0–100 kg), by measuring maximal isometric strength. Patients were first familiarized with the device and then assessed while seated, with both arms bent at a 90º angle at the elbows. They were instructed to hold the dynamometer and squeeze it with maximal effort. Three measurements were taken for each hand, with one-minute intervals between them, and the highest value recorded was used for analysis [19]. Low muscle strength was defined as HGS < 27 kg for men and < 16 kg for women [12].

### Appendicular skeletal muscle mass

Appendicular skeletal muscle mass (ASM, kg) was estimated using the Lee Eqs [20, 21]. ASM = 0.244 × weight (kg) + 7.80 × height (m) + 6.6 × sex − 0.098 × age (years) + ethnicity factor − 3.3, where sex = 1 for men and 0 for women; ethnicity factor: +0 (White), + 1.4 (Black), − 1.2 (Asian); and height is in meters. We calculated ASMI by dividing ASM by height² (kg/m²). Low muscle mass was defined as an appendicular skeletal muscle mass index (ASMI, kg/m²)—calculated by dividing the result of the Lee equation by height squared—of < 8.74 kg/m² for men and < 6.96 kg/m² for women, both determined at the 20th percentile of the sample distribution [22, 23].

### Physical performance

Physical performance was assessed by measuring gait speed (GS, meters/second). Patients were instructed to walk at their usual pace over a 4-meter distance, repeating the test three times with one-minute intervals. The fastest attempt was considered for analysis. Low physical performance was defined as WS ≤ 0.8 m/s [12].

### Definition and diagnosis of sarcopenia

The diagnostic criteria proposed by the EWGSOP2 were used to define sarcopenia [12]. Patients were categorized as having “probable sarcopenia” when low muscle strength was present, “confirmed sarcopenia” when low muscle strength was accompanied by low muscle mass, and “severe sarcopenia” when low muscle strength, low muscle mass, and low physical performance were all present.

### Data collection

The collected data included sociodemographic variables, lifestyle habits, clinical characteristics related to HIV, and quality of life (QoL). The questionnaire used in the interviews is available as Supplementary material 1 (Interview Questionnaires).

## Covariates

### Sociodemographic variables

Sociodemographic variables were collected through interviews. For participants with limited literacy, trained interviewers administered questionnaires verbatim, using standardized prompts to ensure comprehension and minimize interviewer bias. The following were considered: age (in complete years), age group (adults and older individuals aged ≥ 60 years [24], sex (male or female), self-reported race/ethnicity (White, Black, Brown, Asian, or Indigenous), later grouped into “White,” “Brown,” and “Other”; marital status (single, married, widowed, or separated/divorced); employment status (employed or unemployed); educational attainment (“<4 years,” “4–8 years,” “>8–11 years,” and “>11 years”); and socioeconomic status, classified according to the Brazilian Economic Classification Criteria (Brazilian Criteria ABEP) established by the Brazilian Association of Research Companies into A (R$21.826,74), B (R$5.755,23–10.361,48), C (R$1.965,87–3.276,76), and D/E (R$900,60)—which, in US dollars, would be A ($3,838.68), B ($1,012.18–$1,822.28), C ($345.74–$576.29), and D/E ($158.39) [25].

### Lifestyle habits

Lifestyle habits were also collected through interviews and included alcohol consumption (“does not consume,” “occasionally,” “weekly,” “daily/regularly,” or “former drinker”); smoking (“never smoked,” “currently smokes,” or “former smoker”); and physical activity level (PAL), determined using the International Physical Activity Questionnaire (IPAQ) short version for the Brazilian population validated by Matsudo et al. (2001) [26]. PAL was classified according to the World Health Organization’s guidelines on physical activity and sedentary behavior [27] and categorized as “sufficient” for individuals reporting 150 to 300 min of moderate-intensity or 75 to 150 min of vigorous-intensity physical activity per week, and “insufficient” for those who did not meet these criteria [28].

### Clinical characteristics related to HIV

Disease status was classified as asymptomatic (CD4 + T-cell count > 500 cells/mm³), symptomatic (CD4 + T-cell count between 201 and 499 cells/mm³), and AIDS (CD4 + T-cell count ≤ 200 cells/mm³) [29]. CD4 + T-cell count was also categorized as ≤ 200 cells/mm³ or > 200 cells/mm³ and expressed as an absolute number (cells/mm³). The time since diagnosis was calculated in months (the difference between the diagnosis date in the medical chart and the interview date). Time on treatment was categorized as ≤ 6 years or > 6 years, based on the median found in the present study. Opportunistic infections were classified as present or absent.

Antiretroviral therapies were categorized by drug class, considering the use of nucleoside reverse transcriptase inhibitors (NRTIs), non-nucleoside reverse transcriptase inhibitors (NNRTIs), integrase inhibitors (INIs), and protease inhibitors (PIs), with each class categorized as “yes” or “no” [30]. Additionally, therapeutic regimens were categorized as follows: 2 NRTIs + INI, 2 NRTIs + PI, 2 NRTIs + NNRTI, 3 NRTIs + PI, and unspecified ART.

### Quality of life as an outcome for cutoff determination

This study used QoL as an outcome measure to identify the optimal cutoff points for HGS in discriminating individuals with poorer QoL. Perceived QoL was assessed using the Portuguese version of the WHOQOL-HIV Bref instrument [31].

The WHOQOL-HIV Bref is a shortened version of the WHOQOL-HIV developed by the World Health Organization to assess QoL in PLHIV. It was adapted from the WHOQOL-100 [32] and the WHOQOL-Bref [33], incorporating HIV-specific items. The instrument contains 31 items rated on a 5-point Likert scale, evaluating individuals’ perceptions over the previous 15 days. It covers six domains: *physical* (pain, energy, sleep, and fatigue), *psychological* (positive and negative feelings, self-esteem, and body image), *level of independence* (mobility, activities of daily living, and work capacity), *social relationships* (social support and satisfaction with relationships), *environment* (safety, access to healthcare, transportation, and financial resources), *and spirituality/religion/personal beliefs* (importance of faith and life meaning). It also includes two general items related to overall QoL and perceived health status.

Domain scores range from 4 to 20 and are calculated by averaging the responses to the items within each domain. Higher scores indicate better quality of life. In this study, QoL was dichotomized based on the sample median, a strategy previously adopted in the literature [11], to ensure balanced representation of individuals with better and poorer QoL.

### Sample size calculation

The sample size was estimated a priori using the standard formula for cross-sectional studies based on proportion (Eq. 1), given that the main outcome variable (sarcopenia) was binary. This method provides a conservative estimate in the absence of prior data on effect size. Considering a 95% confidence level (Z = 1.96), a 5% margin of error (E = 0.05), and an expected proportion of 8.7% for sarcopenia [8], the minimum sample size estimated was 123 participants.

1 $${\rm{n = }}{{{{\rm{Z}}^{\rm{2}}}{\rm{. p }}{\rm{. }}\left( {{\rm{1 - p}}} \right)} \over {{{\rm{E}}^{\rm{2}}}}}$$

Equation 1. Sample size calculation based on proportion estimates (where *n* represents the sample size, *Z* is the value from the standard normal distribution corresponding to a 95% confidence level [1.96], *p* is the expected prevalence of the condition of interest, and *E* is the acceptable margin of error).

A total of 105 patients were included due to population and logistical constraints. A post hoc power analysis for the main logistic regression model (α = 0.05; observed sarcopenia prevalence = 21.9%) indicated a statistical power of approximately 82%, supporting the adequacy of the achieved sample size for the analyses conducted.

### Statistical analysis

Descriptive and inferential analyses were performed using IBM SPSS Statistics^®^ version 22 (SPSS, an IBM Company, Chicago, IL), with a significance level set at *p* < 0.05. Diagnostic accuracy analyses were performed using MedCalc version 23.1.3.

The Kolmogorov–Smirnov test was applied to assess data normality. Parametric variables were presented as means and standard deviations, and nonparametric variables as medians.

Sensitivity, specificity, positive and negative likelihood ratios (LR + and LR−), areas under the receiver operating characteristic curves (AUC), and Youden’s index were calculated to determine the diagnostic accuracy of HGS in identifying poorer QoL. Cutoff points were defined based on the results obtained in this study.

Sarcopenia was defined using the algorithm proposed by the EWGSOP2. For comparison purposes, two constructs were adopted:

#### Construct 1

low muscle strength defined as HGS < 27 kg for men and < 16 kg for women, and low muscle mass defined as ASMI < 8.74 kg/m² for men and < 6.96 kg/m² for women;

#### Construct 2

low muscle strength based on the cutoff points identified through the diagnostic accuracy analysis conducted in this study. In both constructs, low gait speed was defined as ≤ 0.8 m/s.

Binary logistic regression models were applied to examine the association between independent variables and the outcome (sarcopenia). Prior to regression analysis, sarcopenia prevalence was estimated for both constructs. In both **Construct** 1 and **Construct 2**, participants were categorized as “no sarcopenia” (0) and “probable, confirmed, or severe sarcopenia” (1), due to the small sample size in the confirmed and severe sarcopenia groups. Sex and age were included as adjustment variables, given their association with the outcome. Sarcopenia component variables were excluded due to multicollinearity with the outcome.

Constructs were constructed in three steps:

#### Step 1

The chi-square test was used to identify statistically significant associations (*p* < 0.05). Crude logistic regression models were then developed for each independent variable significantly associated with sarcopenia to explore potentially relevant factors.

#### Step 2

Multiple models were constructed including the variables that were significant in the crude models (when analyzed individually), as well as those with *p* < 0.20, ensuring the absence of multicollinearity and verifying the variance inflation factor (VIF) within acceptable limits.

#### Step 3

The logistic regression model fit quality was evaluated by considering the models’ overall accuracy, sensitivity and specificity, and the Nagelkerke R².

### Ethical procedures

The Research Ethics Committee of the Federal University of Grande Dourados (protocol no. 5.919.928; amendment no. 6.559.968) approved this study. All participants signed an Informed Consent Form (ICF).

## Results

**Fig. 1:**
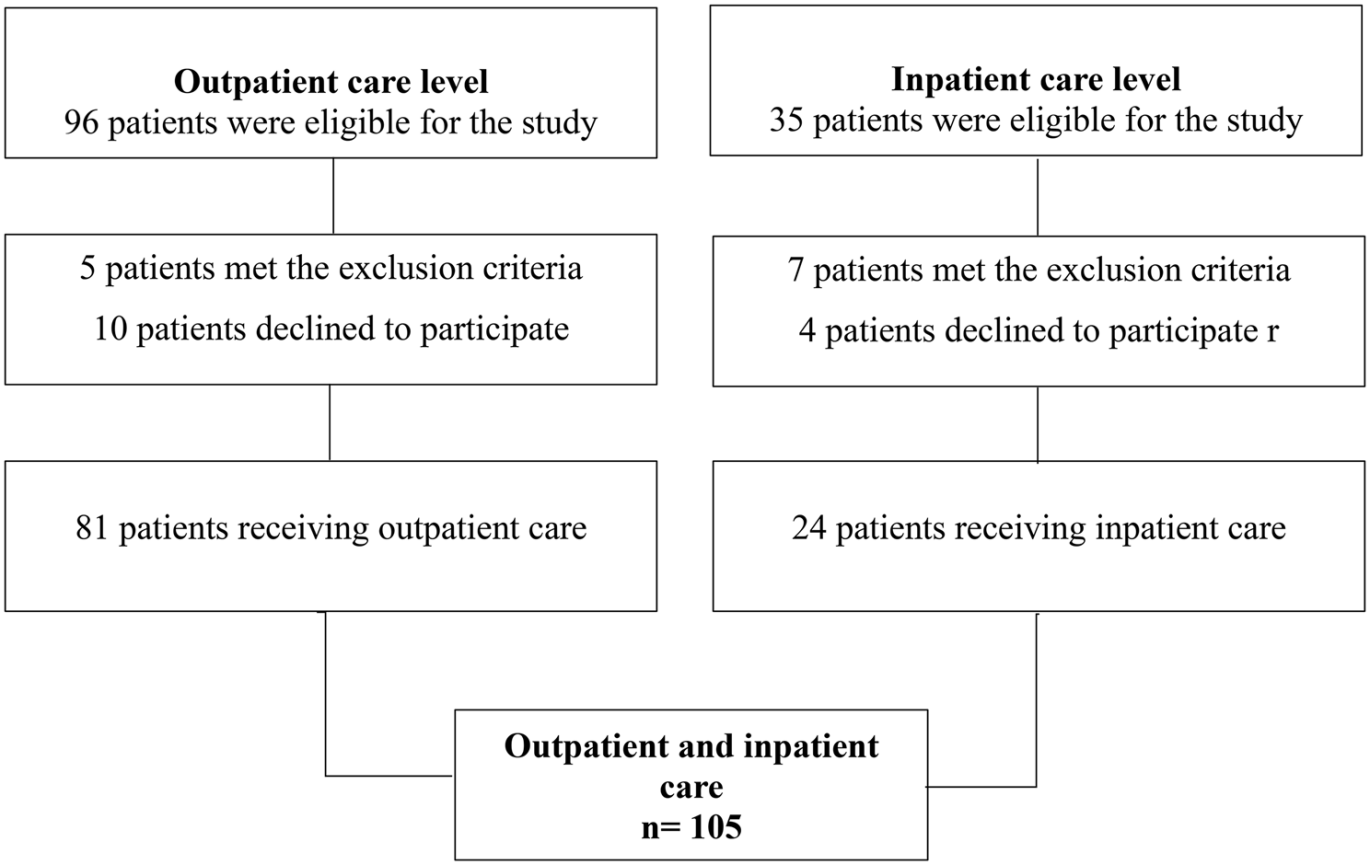
Patient inclusion flowchart

A total of 105 patients were included in the study (Fig. 1). The sample was predominantly composed of men (51.4%), single individuals (43.8%), self-identified as Brown (59.0%), individuals with 4 to 8 years of education (41.9%), belonging to socioeconomic level C (61.0%), and without employment (63.8%). Regarding lifestyle habits, 44.8% reported not consuming alcoholic beverages, 52.4% reported not smoking, and 71.4% reported insufficient physical activity levels. As for clinical characteristics related to PLHIV, 35.2% were symptomatic, 65.7% had CD4 + T-cell counts > 200 cells/mm³, 51.4% had been undergoing treatment for more than 6 years, and 51.4% were receiving care at a specialized outpatient service. Regarding ART types, most patients were using NRTIs (91.4%) (Table 1).

**Table 1:** Sample characteristics (*n* = 105)

Variables		*n*	%
**Age (years)**,** Mean ± SD**	44.6 ± 13.5		
**Body mass index (BMI) (kg/m²)**,** Mean ± SD**	24.1 ± 6.2		
***Sociodemographic variables***			
**Age group (%)**			
Adult		91	86.7
Older individuals		14	13,3
**Sex**			
Male		54	51.4
**Self-reported race/ethnicity (%)**			
Brown		62	59.0
**Marital status (%)**			
Married		34	32.4
Single		46	43.8
Widowed		7	6.7
Separated/Divorced		18	17.1
**Employment status (%)**			
No		67	63.8
**Educational attainment (%)**			
< 4 years		11	10.5
4–8 years		44	41.9
8–11 years		29	27.6
11 years		21	20.0
**Economic level**^**#**^ **(%)**			
C		64	61.0
***Lifestyle habits***			
**Alcohol consumption (%)**			
Does not consume		47	44.8
Occasionally		5	4.8
Weekly		6	5.9
Daily		28	26.7
Former drinker		19	18.1
**Smoking status (%)**			
Never smoked		55	52.4
**Physical activity level (%)**			
Insufficient		75	71.4
***Clinical variables***			
**Disease status (%)**			
Asymptomatic		32	30.5
Symptomatic		37	35.2
AIDS		36	34.3
CD4 + T-cell count*		373.9 ± 292.4 (Med = 342.5)	
> 200 cells/mm³		69	65.7
**Opportunistic infections (%)**			
No		69	65.7
**Time on treatment (months)**		106.8 ± 106.3 (Med = 72.0)	
6 years		54	51.4
**Care setting (%)**			
Outpatient		27	25.7
SAE/CTA		54	51.4
Inpatient		24	22.9
**Types of ART (%)**			
NRTI, yes		96	91.4
INI, yes		75	71.4
**ART regimens (%)**			
2 NRTIs + INI		70	66.7
**SARC-F (%)**			
No risk of sarcopenia		77	73.3
**SARC-Calf (%)**			
No risk of sarcopenia		63	61.2
Sarcopenia components			
**Handgrip strength (HGS)**			
HGS, kg (%)	27.9 ± 10.9		
Men	32.9 ± 11.4		
Women	22.8 ± 7.7		
Low muscle strength (< 27/16 kg)		23	21.9
**Appendicular skeletal muscle mass index (ASMI)**			
**ASMI**,** kg/m²**		9.3 ± 1.6	
Men		10.0 ± 1.4	
Women		8.6 ± 1.7	
Low muscle mass (< 8.74/6.96 kg/m²)		20	19.0
**Physical performance**			
Gait speed (m/s) ^δ^	0.86 ± 0.30		
Men	0.80 ± 0.30		
Women	0.93 ± 0.29		
Low gait speed		45	42.9
**Quality of life**			
Mean overall score		14.4 ± 2.5	
Men		14.5 ± 2.3	
Women		14.3 ± 2.7	
Poor quality of life (score < 14.7)		52	49.5

Note: SD: Standard deviation; # Economic level determined by ABEP: C = R$ 1,965.87 to 3,276.76; Med: Median; AIDS: Acquired immunodeficiency syndrome; * CD4 + T-cell count (*n* = 98); SAE/CTA: Specialized Care Service and Counseling and Testing Center; ART: Antiretroviral therapy; NRTI: Nucleoside reverse transcriptase inhibitor; INI: Integrase inhibitor; δ Gait speed (*n* = 103)

**Fig. 2:**
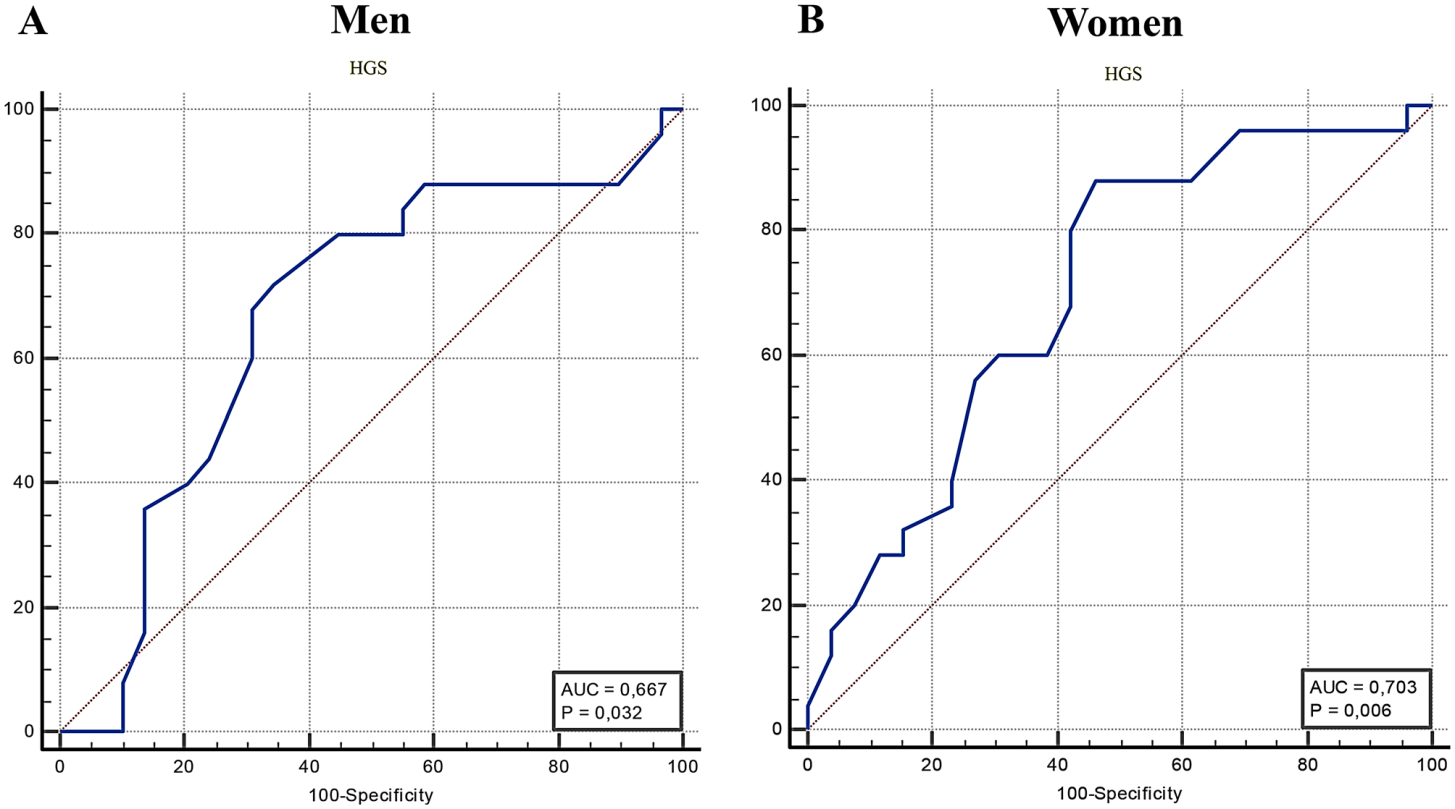
ROC curves of handgrip strength for predicting poorer quality of life in men (**A**) and women (**B**) living with HIV

The HGS cutoff points with the best discriminatory power for poorer QoL were ≤ 33 kg for men and ≤ 26 kg for women, with 72.0% sensitivity and 65.52% specificity for men, and 88.0% sensitivity and 53.85% specificity for women, respectively (Youden’s index: 0.37 and 0.41; AUC: 0.66 and 0.70) (Fig. 2, A and B; Table 2). Based on these cutoff points, Construct 2 was created, defining low muscle strength as HGS ≤ 33/26 kg for men and women, respectively. Supplementary material 2 (Supplementary Table) presents all tested cutoff points for HGS in men and women.

**Table 2:** Diagnostic properties and cutoff points of handgrip strength (HGS) for identifying the quality of life (QoL) outcome

Cutoff points	Sensitivity	95% CI	Specificity	95% CI	LR+	LR-	AUC	95% CI	Youden’s index
**HGS**									
***Men (n = 54)***									
≤ 33	72.00	50.6–87.9	65.52	45.7–82.1	2.09	0.43	0.66	0.51–0.81	0.37
***Women (n = 51)***									
≤ 26	88.00	68.8–97.5	53.85	33.4–73.4	1.91	0.22	0.70	0.55–0.84	0.41

Note: LR+: positive log-likelihood; LR−: negative log-likelihood; AUC: area under curve; HGS: handgrip strength

**Table 3:** Prevalence of sarcopenia according to different sarcopenia constructs

Prevalence, *n* (%)		
Sarcopenia status	Sarcopenia construct	
	Construct 1	Construct 2
No sarcopenia	82 (78.1)	43 (41.0)
Probable sarcopenia	14 (13.3)	45 (42.9)
Confirmed or severe sarcopenia	9 (8.6)	17 (16.2)

Note: Construct 1: < 27/16 kg, < 8.74/6.96 kg/m^2^, ≤ 0.8 m/s; Construct 2: ≤ 33/26 kg, < 8.74/6.96 kg/m^2^, ≤ 0.8 m/s. Confirmed and severe sarcopenia were analyzed as a single category, in accordance with the EWGSOP2 algorithm. Probable sarcopenia was analyzed as a separate category. Sarcopenia constructs differed only by the handgrip strength thresholds applied

The prevalence of sarcopenia (confirmed and severe) was 8.6% in Construct 1 and 16.2% in Construct 2 (Table 3)

Table 4 shows that CC, QoL, and the sarcopenia components—muscle strength and physical performance—were associated with both Construct 1 and Construct 2.

**Table 4:** Factors associated with sarcopenia

	Construct 1			Construct 2		
	No sarcopenia *n* = 82	Sarcopenia *n* = 23		No sarcopenia *n* = 43	Sarcopenia *n* = 62	
Variables	*n* (%)	*n* (%)	*p*-value	*n* (%)	*n* (%)	*p*-value
***Sociodemographic variables***						
**Age group**			1,000			0,148
Adult	71 (86,6)	20 (87,0)		40 (93,0)	51 (82,3)	
Older individual	11 (13,4)	3 (13,0)		3 (7,0)	11 (17,7)	
**Sex**			0,642			0,165
Male	41 (50,0)	13 (56,5)		26 (60,5)	28 (45,2)	
Female	41 (50,0)	10 (43,5)		17 (39,5)	34 (54,8)	
**Self-reported race/ethnicity**			**0**,002*			0,189
White	17 (20,7)	13 (56,5)		9 (20,9)	21 (33,9)	
Non-White	65 (79,3)	10 (43,5)		34 (79,1)	41 (66,1)	
**Marital status**			0,206			0,112
Single	32 (39,0)	14 (60,9)		21 (48,8)	25 (40,3)	
Married	28 (34,1)	6 (26,1)		13 (30,2)	21 (33,9)	
Widowed	7 (8,5)	0 (0,0)		-	7 (11,3)	
Divorced	15 (18,3)	3 (13,0)		9 (20,9)	9 (14,5)	
**Employment status**			1,000			0,098
No	52 (63,4)	15 (65,2)		23 (53,5)	44 (71,0)	
Yes	30 (36,6)	8 (34,8)		20 (46,5)	18 (29,0)	
**Educational attainment**			0,825			0.140
< 4 years	8 (9.8)	3 (13.0)		3 (7.0)	8 (12.9)	
4–8 years	33 (40.2)	11 (47.8)		16 (37.2)	28 (45.2)	
8–11 years	24 (29.3)	5 (21.7)		17 (39.5)	12 (19.4)	
11 years	17 (20.7)	4 (17.4)		7 (16.3)	14 (22.6)	
**Economic level** ^**#**^			0.062			0.512
A	1 (1.2)	-		1 (2.3)	-	
B	22 (26.8)	2 (8.7)		11 (25.6)	13 (21.0)	
C	50 (61.0)	14 (60.9)		26 (60.5)	38 (61.3)	
D/E	9 (11.0)	7 (30.4)		5 (11.6)	11 (17.7)	
***Lifestyle habits***						
**Alcohol consumption**			0.902			0.846
Does not consume	37 (45.1)	10 (43.5)		18 (41.9)	29 (46.8)	
Occasionally	4 (4.9)	1 (4.3)		3 (7.0)	2 (3.2)	
Weekly	4 (4.9)	2 (8.7)		2 (4.7)	4 (6.5)	
Daily/regularly	21 (25.6)	7 (30.4)		11 (25.6)	17 (27.4)	
Former drinker	16 (19.5)	3 (13.0)		9 (20.9)	10 (16.1)	
**Smoking status**			0.353			0.156
Never smoked	46 (56.1)	9 (39.1)		27 (62.8)	28 (45.2)	
Currently smokes	15 (18.3)	8 (34.8)		8 (18.6)	13 (21.0)	
Former smoker	21 (25.6)	6 (26.1)		8 (18.6)	21 (33.9)	
**Physical activity level**			0.204			1.000
Insufficient	56 (68.3)	19 (82.6)		31 (72.1)	44 (71.0)	
Sufficient	26 (31.7)	4 (17.4)		12 (27.9)	18 (29.0)	
***Clinical variables***						
**Disease status**			0.004*			0.138
Asymptomatic	30 (36.6)	2 (8.7)		15 (34.9)	17 (27.4)	
Symptomatic	30 (36.6)	7 (30.4)		18 (41.9)	19 (30.3)	
AIDS	22 (26.8)	14 (60.9)		10 (23.3)	26 (41.9)	
**Opportunistic infections**			0.624			0.534
No	55 (67.1)	14 (60.9)		30 (69.8)	39 (62.9)	
Yes	27 (32.9)	9 (39.1)		13 (30.2)	23 (37.1)	
**CD4 + T-cell count** ^**δ**^			0.005*			0.061
≤ 200 cells/mm³	22 (26.8)	14 (60.9)		10 (23.3)	26 (41.9)	
> 200 cells/mm³	60 (73.2)	9 (39.1)		33 (76.7)	36 (58.1)	
**Time on treatment (complete years)**			0.239			0.964
≤ 6 years	37 (45.1)	14 (60.9)		21 (48.8)	30 (48.4)	
6 years	45 (54.9)	9 (39.1)		22 (51.2)	32 (51.6)	
**Care setting**			< 0.001*			0.054
Inpatient	11 (13.4)	13 (56.5)		6 (14.0)	18 (29.0)	
Outpatient	23 (28.0)	4 (17.4)		9 (20.9)	18 (29.0)	
SAE/CTA	48 (58.5)	6 (26.1)		28 (65.1)	26 (41.9)	
**Types of ART**						
NRTI			0.407			0.734
Yes	76 (92.7)	20 (87.0)		3 (7.0)	6 (9.7)	
No	6 (7.3)	3 (13.0)		40 (93.0)	56 (90.3)	
NNRTI			0.335			1.000
Yes	6 (7.3)	-		41 (95.3)	58 (93.5)	
No	76 (92.7)	23 (100)		2 (4.7)	4 (6.5)	
INI			1.000			0.189
Yes	58 (70.7)	17 (73.9)		9 (20.9)	21 (33.9)	
No	24 (29.3)	6 (26.1)		34 (79.1)	41 (66.1)	
PI			0.553			0.620
Yes	17 (20.7)	3 (13.0)		36 (83.7)	49 (79.0)	
No	65 (79.3)	20 (87.0)		7 (16.3)	13 (21.0)	
**ART regimens**			0.470			0.667
Unspecified ART	6 (7.3)	3 (13.0)		3 (7.0)	6 (9.7)	
2 NRTIs + INI	53 (64.6)	17 (73.9)		31 (72.1)	39 (62.9)	
2 NRTIs + PI	10 (12.2)	3 (13.0)		3 (7.0)	10 (16.1)	
2 NRTIs + NNRTI	6 (7.3)	-		2 (4.7)	4 (6.5)	
2 NRTIs + INI + PI	5 (6.1)	-		3 (7.0)	2 (3.2)	
3 NRTIs + PI	2 (2.4)	-		1 (2.3)	1 (1.6)	
**Anthropometry**						
**CC (cm)** ^†^			< 0.001*			< 0.001*
Adequate	47 (58.8)	3 (13.0)		30 (69.8)	20 (33.3)	
Low muscle mass	33 (41.3)	20 (87.0)		13 (30.2)	40 (66.7)	
***Risk of sarcopenia***						
**SARC-F**			< 0.001*			**0.001**
No risk of sarcopenia	68 (82.9)	9 (39.1)		39 (90.7)	38 (61.3)	
Risk of sarcopenia	14 (17.1)	14 (60.9)		4 (9.3)	24 (38.7)	
**SARC-Calf**			< 0.001*			< 0.001*
No risk of sarcopenia	58 (72.5)	5 (21.7)		35 (81.4)	28 (46.7)	
Risk of sarcopenia	22 (27.5)	18 (78.3)		8 (18.6)	32 (53.3)	
***Sarcopenia components***						
**HGS**						
HGS < 27/16 kg			< 0.001*			< 0.001*
Adequate	82 (100.0)	-		43 (100.0)	39 (62.9)	
Low muscle strength	-	23 (100.0)		-	23 (37.1)	
HGS ≤ 33/26 kg			< 0.001*			< 0.001*
Adequate	43 (52.4)	-		43 (100.0)	-	
Low muscle strength	39 (47.6)	23 (100.0)		-	62 (100.0)	
**ASMI**						
ASMI < 8.74/6.96 kg/m²			0.013*			0.775
Adequate	71 (86.6)	14 (60.9)		38 (88.4)	53 (85.5)	
Low muscle mass	11 (13.4)	9 (39.1)		5 (11.6)	9 (14.5)	
**Gait speed ** ^**γ**^			< 0.001*			**0.005**
Adequate	55 (67.1)	5 (21.7)		32 (74.4)	28 (45.2)	
Low gait speed	27 (32.9)	18 (78.3)		11 (25.6)	34 (54.8)	
**Quality of life**			0.035*			< 0.001*
Better QoL	46 (56.1)	7 (30.4)		31 (72.1)	22 (35.5)	
Poorer QoL	36 (43.9)	16 (69.6)		12 (27.9)	40 (64.5)	

Note: **Construct 1**: <27/16 kg, < 8.74/6.96 kg/m², ≤ 0.8 m/s; **Construct 2**: ≤33/26 kg, < 8.74/6.96 kg/m², ≤ 0.8 m/s; *Statistically significant difference (*p* < 0.05); # Economic level determined by ABEP: A = R5,755.23–10,361.48; C = R900.60; δ CD4 + T-cell count (*n* = 98); † Calf circumference (*n* = 103); γ Gait speed (*n* = 103); ART: Antiretroviral therapy; NRTI: Nucleoside reverse transcriptase inhibitor; NNRTI: Non-nucleoside reverse transcriptase inhibitor; INI: Integrase inhibitor; PI: Protease inhibitor; CC: Calf circumference; HGS: Handgrip strength; ASMI: Appendicular skeletal muscle mass index; QoL: Quality of life

Table 5 presents the logistic regression models using Constructs 1 and 2 as dependent variables. During analysis, collinearity was observed between the independent variables disease status and CD4 + T-cell count, as well as between CC and care setting (VIF > 5), possibly because hospitalized patients may have reduced CC. Only CD4 + T-cell count was included in the model to minimize this effect, as both variables are clinically relevant in the context of HIV.

In the adjusted model using Construct 1, individuals with CD4 + T-cell counts ≤ 200 cells/mm³ had approximately four times greater odds of sarcopenia than the reference group (OR = 4.09; 95% CI: 1.14–14.73; *p* = 0.031). Hospitalized patients had higher odds of developing sarcopenia than those receiving outpatient care at HU or SAE (OR = 8.84; 95% CI: 2.44–32.11; *p* = 0.001). In the same model, poorer QoL was not significantly associated with the outcome.

In the adjusted model using Construct 2, immunosuppression was also associated with approximately fourfold greater odds of sarcopenia (OR = 4.07; 95% CI: 1.29–12.88; *p* = 0.017). Although hospitalization was significant in the unadjusted model, it remained insignificant after adjustment (OR = 2.89; 95% CI: 0.79–10.55; *p* = 0.109). In this model, poorer QoL was significantly associated with sarcopenia: patients with median QoL scores < 14.7 points had approximately five times greater odds of sarcopenia than those with better QoL (OR = 5.12; 95% CI: 2.02–12.98; *p* = 0.001).

The adjusted model based on Construct 1 showed higher specificity (91.5%) and overall accuracy (81.0%), but lower sensitivity (43.5%) and a Nagelkerke R² of 0.337. In contrast, the adjusted model based on Construct 2 demonstrated higher sensitivity (79.0%), specificity of 62.8%, overall accuracy of 72.4%, and a Nagelkerke R² of 0.322. Broader confidence intervals were observed in Construct 1.

**Table 5:** Logistic regression models using Constructs 1 and 2 as dependent variables

Construct 1	Crude model		Multiple model		Adjusted model	
	OR (95% CI)	*p*-value	OR (95% CI)	*p*-value	OR (95% CI)	*p*-value
*CD4 + T-cell count*						
> 200 cells/mm^3^	1.00		1.00		1.00	
≤ 200 cells/mm^3^	4.24 (1.61–11.18)	**0.003**	2.54 (0.85–7.60)	0.094	4.09 (1.14–14.73)	**0.031**
*Care setting*						
Outpatient care (HU e SAE)	1.00		1.00		1.00	
Inpatient care (HU)	8.39 (2.96–23.75)	**< 0.001**	5.74 (1.88–17.50)	**0.002**	8.84 (2.44–32.11)	**0.001**
*QoL*						
≥ 14.7, median score	1.00		1.00		1.00	
< 14.7, median score	2.92 (1.09–7.86)	**0.034**	2.66 (0.89–7.98)	0.081	2.67 (0.86–8.27)	0.088
	**Crude model**		**Multiple model**		**Adjusted model**	
**Construct 2**	**OR (95% CI)**	**p-value**	**OR (95% CI)**	**p-value**	**OR (95% CI)**	**p-value**
*CD4 + T-cell count*						
> 200 cells/mm^3^	1.00		1.00		1.00	
≤ 200 cells/mm^3^	2.38 (1.00-5.68)	0.050	1.98 (0.74–5.34)	0.175	4.07 (1.29–12.88)	**0.017**
*Care setting*						
Outpatient care (HU e SAE)	1.00		1.00		1.00	
Inpatient care (HU)	2.52 (0.91–7.01)	0.076	1.61 (0.51–5.12)	0.421	2.89 (0.79–10.55)	0.109
*QoL*						
≥ 14.7, median score	1.00		1.00		1.00	
< 14.7, median score	4.70 (2.02–10.94)	**< 0.001**	4.46 (1.88–10.58)	**0.001**	5.12 (2.02–12.98)	**0.001**

Note: OR: Odds Ratio; SAE: Specialized Care Service; HU: University Hospital; CC: Calf circumference; QoL: Quality of life; Adjusted model: adjusted for sex and age. **Construct 1**: <27/16 kg, < 8.74/6.96 kg/m², ≤ 0.8 m/s; **Construct 2**: ≤33/26 kg, < 8.74/6.96 kg/m², ≤ 0.8 m/s. Bold = statistically significant

## Discussion

This study identified that handgrip strength cutoff points of ≤ 33 kg for men and ≤ 26 kg for women demonstrated greater diagnostic accuracy for poorer QoL in PLHIV. When the exploratory handgrip strength thresholds established in this study (Construct 2) were applied, a larger proportion of participants were classified as having low muscle strength and sarcopenia according to the EWGSOP2 algorithm, reflecting greater sensitivity in a screening context for identifying early functional vulnerability and quality-of-life impairment, rather than a true increase in sarcopenia prevalence. As reported in the literature, using the EWGSOP2 algorithm and its proposed cutoff points may underestimate the prevalence of sarcopenia in PLHIV [4, 5, 8, 9, 13]. In both the general population and among PLHIV, the definition of sarcopenia and the thresholds for low muscle strength have been widely debated in the scientific literature, with evidence suggesting that EWGSOP2 serves as a good predictor of adverse outcomes only when higher HGS cutoff points are applied [34, 35]. As observed in this study, higher handgrip strength thresholds may be more suitable for the early identification of functional vulnerability and quality-of-life impairment in this population.

Regarding associations, CD4 + T-cell count was associated with sarcopenia regardless of the cutoff points applied (Constructs 1 and 2). Although the relationship between sarcopenia and CD4 + T-cell count in PLHIV is not yet fully understood, existing evidence suggests a possible association between these two factors [6, 36, 37]. Individuals with lower CD4 + T-cell counts tend to present with greater levels of low-grade systemic inflammation and heightened immune activation, which contribute to muscle mass loss and the development of sarcopenia. A CD4 + T-cell count ≤ 200 cells/mm³, combined with the presence of opportunistic infections, characterizes the AIDS stage, which is also associated with an increased risk of sarcopenia [36].

Hospitalized patients had higher odds of sarcopenia when assessed using the < 27 kg (men) and < 16 kg (women) cutoff points for low muscle strength than those receiving outpatient care. These thresholds appear more effective in more severe clinical settings, such as hospitalization, where sarcopenia tends to be more prevalent [8]. Conversely, the cutoff points of ≤ 33/26 kg showed better performance in outpatient screening, enabling earlier identification of functional vulnerability and sarcopenia-related impairment and allowing for the implementation of preventive strategies.

Hospitalization is frequently associated with prolonged immobility, malnutrition, metabolic stress, and increased energy demands—factors significantly contributing to the loss of muscle mass and strength. Hospitalized patients tend to reduce their food intake due to feeding difficulties, lack of appetite, or dietary restrictions imposed by treatment. Reduced protein and caloric intake compromises the maintenance of muscle mass and exacerbates sarcopenia. Moreover, unlike outpatients, hospitalized individuals often spend extended periods confined to bed, leading to rapid muscle wasting [38, 39]. In this context, hospitalization may serve as an indicator of worse clinical status and increased vulnerability to sarcopenia in this population.

Among PLHIV, the reason for hospitalization may be a key factor in understanding the association between inpatient care and the increased likelihood of sarcopenia. A study by Almeida et al. [5], which investigated factors associated with sarcopenia in hospitalized PLHIV, found that 89% of hospitalizations were due to clinical causes, most of which (59%) were related to opportunistic infections. It is well established that poor adherence to treatment can lead to the development of opportunistic infections, resulting in greater debilitation and a higher risk of hospitalization. These combined factors contribute to muscle mass loss and reduced physical performance during hospitalization, promoting the onset of sarcopenia [39].

QoL scores below the median (14.7 points) also predicted sarcopenia in our sample, suggesting that poorer perceived QoL may be associated with greater muscle decline and functional impairment in PLHIV. This finding is consistent with previous studies demonstrating an inverse relationship between sarcopenia and QoL [40, 41]. In a study by Chagas et al. [40], older adults with sarcopenia had significantly lower QoL scores in the domains of physical function, bodily pain, general health, and social functioning. After adjustments, sarcopenia remained inversely associated with physical functioning and general health status. These results suggest that poor QoL may worsen the progression of sarcopenia, as it is linked to the gradual loss of muscle strength and mass, resulting in functional limitations, increased pain perception, and greater social impairment [40]. Furthermore, factors such as social stigma, the presence of comorbidities, lack of emotional support, and physical limitations negatively affect the QoL of PLHIV, thereby increasing the risk of developing and progressing sarcopenia [42]. In this context, the assessment of QoL in PLHIV warrants special attention, as functional and psychosocial dimensions influence the course of sarcopenia.

It is important to emphasize that quality of life in PLHIV is a multifactorial construct influenced by clinical status, psychosocial factors, mental health, stigma, social support, and access to care. Therefore, reduced quality of life cannot be attributed exclusively to sarcopenia or muscle function decline. In this context, handgrip strength should be interpreted as a functional marker associated with vulnerability and impaired quality of life, rather than as a direct or isolated determinant.

We deemed important to highlight the strengths and limitations of this study. One notable strength is that this is the first study to propose specific cutoff points for low muscle strength in PLHIV. These thresholds are particularly relevant because they may facilitate the early identification of functional decline and sarcopenia in this population, potentially supporting timely clinical interventions.

One study limitation was the use of a predictive equation to estimate muscle mass. Although this approach has lower accuracy compared with reference imaging methods, such as dual-energy X-ray absorptiometry (DXA), it represents a feasible and accessible alternative in settings where high-cost equipment is unavailable, which is common in public health services. In PLHIV, chronic inflammation, immune dysfunction, and body composition alterations may affect the accuracy of anthropometric prediction equations [43]. Therefore, it is plausible that the use of the Lee equation [20] may have led to an underestimation of muscle mass and, consequently, of sarcopenia prevalence in our sample, rather than an overestimation.

The Lee equation demonstrated good agreement with DXA in its original validation in adult populations and has since been applied in epidemiological studies and in clinical settings involving chronic conditions, particularly when direct assessment of body composition is not feasible [20, 44–46]. In the context of HIV care, where access to imaging techniques is often limited, the use of predictive equations remains a pragmatic approach for outpatient and large-scale assessments. Nevertheless, future studies should prioritize direct measures of muscle mass to validate and refine sarcopenia assessment in people living with HIV. A further limitation concerns the cross-sectional design, which precludes causal inferences regarding the observed associations.

Additionally, the cutoff points were determined based on an outcome that may vary over time. QoL is a dynamic measure influenced by multiple clinical, emotional, and social factors that may fluctuate over the course of the disease. This variability may limit the generalizability and long-term stability of the proposed HGS thresholds, and caution is warranted when extrapolating these findings. Additionally, treatment adherence to ART was not directly assessed in this study. The sample included both hospitalized and outpatient PLHIV, many of whom were in advanced stages of infection. The high proportion of participants classified as having AIDS and presenting with low CD4 + T-cell count likely reflects the clinical characteristics of hospitalized patients, who are often admitted due to opportunistic infections or complications associated with irregular or interrupted ART use. Therefore, it is plausible that some participants had suboptimal or inconsistent adherence prior to data collection. Finally, the study did not include a disease-specific quality of life instrument, such as the SarQoL. Future investigations should consider incorporating this validated questionnaire to provide a more precise and comprehensive assessment of sarcopenia-related quality of life.

Nonetheless, we adopted rigorous outcome selection criteria to mitigate these limitations, ensured proper training and calibration of the data collection team, and used validated instruments.

## Conclusion

Handgrip strength (HGS) thresholds of ≤ 33 kg for men and ≤ 26 kg for women for defining low muscle strength showed better performance in identifying poorer quality of life in people living with HIV and may be useful for early outpatient screening of functional vulnerability associated with sarcopenia in this population. Conventional thresholds (< 27/16 kg) may better reflect more advanced or severe clinical conditions. In both models, sarcopenia was associated with immunosuppression (CD4 + T cell count ≤ 200 cells/mm³). These findings highlight the potential relevance of population-specific criteria for improving early detection and clinical management, although results should be interpreted as exploratory and considered in conjunction with clinical judgment.

## Supplementary Information

Below is the link to the electronic supplementary material.

Supplementary Material 1

Supplementary Material 2

## Data Availability

The data supporting the conclusions of this article are not publicly available in order to protect patient confidentiality. However, access to the data may be granted upon reasonable request to the corresponding author.

## Abbreviations

AIDS: Acquired Immunodeficiency Syndrome
AUC: Area Under the Curve
CP: Calf Circumference
EWGSOP2: European Working Group on Sarcopenia in Older People
HGS: Handgrip Strength
HIV: Human Immunodeficiency Virus
HU: University Hospital
HU-UFGD: University Hospital of the Federal University of Grande Dourados (HU-UFGD)
BMI: Body Mass Index
CI: Confidence Interval
ASMI: Appendicular Skeletal Muscle Mass Index
INI: Integrase Inhibitor
PI: Protease Inhibitor
IPAQ: International Physical Activity Questionnaire
STI: Sexually Transmitted Infection
NRTI: Nucleoside Reverse Transcriptase Inhibitor
NNRTI: Non-Nucleoside Reverse Transcriptase Inhibitor
LR: Log-Likelihood Ratio
ASM: Appendicular Skeletal Muscle
PAL: Physical Activity Level
WHO: World Health Organization
OR: Odds Ratio
PLHIV: People Living with HIV
QoL: Quality of Life
SAE/CTA: Specialized Care Service/Testing and Counseling Center
ART: Antiretroviral Therapy
ICF: Informed Consent Form
GS: Gait Speed
WHOQOL-HIV Bref: World Health Organization Quality of Life-HIV Bref
