# Supplementary material for: Factors associated with sarcopenia and exploratory thresholds for low muscle strength in people living with the human immunodeficiency virus
Source: BMC Infect Dis. 2026 Mar 24;26:875. doi: 10.1186/s12879-026-12961-z (PMC13137641; doi:10.1186/s12879-026-12961-z)
Supplement: Supplementary file 1 — Supplementary Material 1 [file 12879_2026_12961_MOESM1_ESM.docx]

**Supplementary material 1**

**Supplementary material.** Interview Questionnaires

**Interview Date: ___/ ___/ ______ ID: __________**

[ ] HU Inpatient  [ ] HU Outpatient Clinic [ ] SAE

**Section I - Sociodemographic Data Collection Form**

**Sex:** ( ) Male ( ) Female            **Date of Birth:** ___/ ___/ ______
**Medical Record Number:** __________________   **Age:** ______ full years
**Employment Status:** ( ) Employed ( ) Unemployed
**Marital Status:** ( ) Single ( ) Married ( ) Widowed ( ) Separated/Divorced
**Self-Declared Race/Color:** ( ) White ( ) Black ( ) Brown (Mixed) ( ) Asian ( ) Prefer not to disclose
**Education Level:** ( ) <4 years ( ) 4–8 years ( ) >8–11 years ( ) >11 years

**Economic Level (Brazilian Economic Classification Criteria – ABEP 2022):**

| Ownership of Household Items | Number of Items | | | | |
| --- | --- | --- | --- | --- | --- |
|  | **0** | **1** | **2** | **3** | **4 ou +** |
| Bathrooms |  |  |  |  |  |
| Domestic Workers (monthly employed) |  |  |  |  |  |
| Automobiles |  |  |  |  |  |
| Personal Computers |  |  |  |  |  |
| Dishwasher |  |  |  |  |  |
| Refrigerator |  |  |  |  |  |
| Freezer |  |  |  |  |  |
| Washing Machine |  |  |  |  |  |
| DVD Player |  |  |  |  |  |
| Microwave Oven |  |  |  |  |  |
| Motorcycle |  |  |  |  |  |
| Clothes Dryer |  |  |  |  |  |

**Educational Level of the Head of Household and Access to Public Services**

( ) Illiterate / Incomplete Primary Education (Early Years)
( ) Complete Primary (Early Years) / Incomplete Lower Secondary
( ) Complete Lower Secondary / Incomplete Upper Secondary
( ) Complete Upper Secondary / Incomplete Higher Education
( ) Complete Higher Education

**Public Services:** **Piped Water: (** ) No ( ) Yes **Paved Street: (** ) No ( ) Yes

**Section II - Lifestyle Habits**

**Alcohol Consumption:**

( ) No, never drank ( ) Yes, regularly ( ) No, drank in the past (former drinker)

**Smoking Habit:**
( ) No, never smoked ( ) Yes, regularly ( ) No, smoked in the past (former smoker)

**Physical Activity Level (IPAQ – International Physical Activity Questionnaire):**

| **IPAQ – International Physical Activity Questionnaire – Short Form**  Matsudo S, Araujo T, Matsudo V, Andrade D. Questionário Internacional de Atividade Física (IPAQ): Estudo de Validade e Reprodutibilidade no Brasil. 2001. |
| --- |
| **We are interested in understanding what types of physical activity people do as part of their daily lives. The following questions relate to the amount of time you spent doing physical activities during the LAST 7 days.** These questions include activities you do at work, for transportation, during leisure time, sports, exercise, or household and gardening tasks. **Your answers are VERY important. Please answer every question, even if you think you are not physically active. Thank you for your participation!**  To answer the questions, please keep in mind that:   - **VIGOROUS physical activities** are those that require a great deal of physical effort and make you breathe MUCH harder than normal. - **MODERATE physical activities** are those that require some physical effort and make you breathe A LITTLE harder than normal.   When answering the questions, consider only the activities that you performed for **at least 10 continuous minutes** at a time. |
| **1a.** On how many days during the last week did you **walk for at least 10 continuous minutes** at home or at work, for transportation (to get from one place to another), for leisure, enjoyment, or as a form of exercise? **Days:** _____ per WEEK ( ) None  **1b.** On the days that you walked for at least 10 continuous minutes, **how much total time** did you usually spend walking per day? **Hours:** ______ **Minutes:** _____ |
| **2a.** On how many days during the last week did you do **MODERATE physical activities** for at least 10 continuous minutes, such as light cycling, swimming, dancing, light aerobic exercise, playing recreational volleyball, carrying light loads, or doing household chores in the house, yard, or garden like sweeping, vacuuming, gardening, or any activity that caused a **moderate increase in your breathing or heart rate**? (**PLEASE DO NOT INCLUDE WALKING**) **Days:** _____ per WEEK ( ) None  **2b.** On the days that you did these moderate activities for at least 10 continuous minutes, **how much total time** did you usually spend doing them per day? **Hours:** ______ **Minutes:** _____ |
| **3a.** On how many days during the last week did you do **VIGOROUS physical activities** for at least 10 continuous minutes, such as running, doing aerobic exercises, playing soccer, fast cycling, playing basketball, doing heavy household chores in the house or yard, digging in the garden, lifting heavy loads, or any activity that caused a **significant increase in your breathing or heart rate**? **Days:** _____ per WEEK ( ) None  **3b.** On the days that you did these vigorous activities for at least 10 continuous minutes, **how much total time** did you usually spend doing them per day? **Hours:** ______ **Minutes:** _____ |
| **The following questions refer to the time you spend sitting each day**—whether at work, school or university, at home, or during your free time. This includes time spent sitting while studying, relaxing, doing homework, visiting friends, reading, or lying down while watching TV. **Do not include time spent sitting while traveling by bus, train, subway, or car.**  **4a.** How much total time do you usually spend **sitting on a weekday**? ______ hours ______ minutes  **4b.** How much total time do you usually spend **sitting on a weekend day**? ______ hours ______ minutes |

**Section III - Clinical Questionnaire**

**Clinical Diagnosis:** ________________________**Year of Positive Test:** _______________
**Current Disease Status:** ( ) Asymptomatic ( ) Symptomatic ( ) AIDS
**Time Since Diagnosis:** ___________**CD4 Count:** ___________
**Previous Chronic Conditions:** ________________________________________________________

Type of Treatment:
( ) ART (Antiretroviral Therapy) ( ) Other: __________________________________

**Medications and/or Supplements in Use *(please list active ingredient, dosage, and frequency)*:**

| **Active Ingredient / Supplement** | **Dosage** | **Frequency** |
| --- | --- | --- |
|  |  |  |
|  |  |  |

**Section IV - Anthropometry, Body Composition, and Physical Tests**

**Weight (kg):** _____ **Height (m):** _____**Arm Circumference (AC) (cm):** ______ **Triceps Skinfold Thickness (TSF) (mm):** ______**Calf Circumference (CC) (cm):** ______

**Adductor Pollicis Muscle Thickness (APMT) (mm):** **Right:** ______ **Left:** ______

**Handgrip Strength (kg/f):**
 **Dominant Hand:**

- 1st Measurement: ______ 2nd Measurement: ______ 3rd Measurement: ______
  **Non-Dominant Hand:**
- 1st Measurement: ______ 2nd Measurement: ______ 3rd Measurement: ______

**Gait Speed Test (seconds):**

- 1st Measurement: ______ 2nd Measurement: ______ 3rd Measurement: ______

**Section V - Sarcopenia Risk Assessment (SARC-F Questionnaire)**

**1. Strength**
How much difficulty do you have in lifting and carrying 10 pounds?
[ ] None  [ ] Some  [ ] A lot or unable

**2. Assistance in walking**
How much difficulty do you have walking across a room?
[ ] None  [ ] Some  [ ] A lot or unable

**3. Rise from a chair**
How much difficulty do you have transferring from a chair or bed?
[ ] None  [ ] Some  [ ] A lot or unable

**4. Climb stairs**
How much difficulty do you have climbing a flight of 10 stairs?
[ ] None  [ ] Some  [ ] A lot or unable

**5. Falls**
How many times have you fallen in the past year?
[ ] None  [ ] 1–3 falls  [ ] 4 or more falls

**Section VI - Quality of Life Assessment**

**WHOQOL-HIV BREF – World Health Organization Quality of Life – HIV Instrument, Short Form**

Original version: https://www.who.int/publications/i/item/WHO-MSD-MER-Rev-2012-02

Validation to Portuguese: Zimpel, R. R., & Fleck, M. P. (2007). Quality of life in HIV-positive Brazilians: application and validation of the WHOQOL-HIV, Brazilian version. AIDS care, 19(7), 923–930. https://doi.org/10.1080/09540120701213765
