# Supplementary material for: Factors associated with sarcopenia and exploratory thresholds for low muscle strength in people living with the human immunodeficiency virus
Source: BMC Infect Dis. 2026 Mar 24;26:875. doi: 10.1186/s12879-026-12961-z (PMC13137641; doi:10.1186/s12879-026-12961-z)
Supplement: Supplementary file 2 — Supplementary Material 2 [file 12879_2026_12961_MOESM2_ESM.docx]

**Supplementary material 2**

**Supplementary Table**. Diagnostic properties and cutoff points of handgrip strength (HGS) for identifying the quality of life (QoL) outcome

| **Cutoff points** | **Sensitivity (%)** | **95% CI Sensitivity** | **Specificity (%)** | **95% CI Specificity** | **LR+** | **95% CI LR+** | **LR-** | **95% CI LR-** |  |
| --- | --- | --- | --- | --- | --- | --- | --- | --- | --- |
| **Men** |  |  |  |  |  |  |  |  |  |
| ≤20 | 4,0 | 0,1 - 20,4 | 89,7 | 72,6 - 97,8 | 0,39 | 0,92 - 1,24 | 1,07 | 1,07 - 1,24 |  |
| ≤21 | 8,0 | 1,0 - 26,0 | 89,7 | 72,6 - 97,8 | 0,77 | 0,87 - 1,22 | 1,03 | 1,03 - 1,22 |  |
| ≤22 | 16,0 | 4,5 - 36,1 | 86,2 | 68,3 - 96,1 | 1,16 | 0,78 - 1,22 | 0,97 | 0,97 - 1,22 |  |
| ≤24 | 24,0 | 9,4 - 45,1 | 86,2 | 68,3 - 96,1 | 1,74 | 0,68 - 1,15 | 0,88 | 0,88 - 1,15 |  |
| ≤26 | 36,0 | 18,0 - 57,5 | 86,2 | 68,3 - 96,1 | 2,61 | 0,53 - 1,03 | 0,74 | 0,74 - 1,03 |  |
| ≤28 | 40,0 | 21,1 - 61,3 | 79,3 | 60,3 - 92,0 | 1,93 | 0,52 - 1,10 | 0,76 | 0,76 - 1,10 |  |
| ≤29 | 44,0 | 24,4 - 65,1 | 75,9 | 56,5 - 89,7 | 1,82 | 0,49 - 1,11 | 0,74 | 0,74 - 1,11 |  |
| ≤30 | 60,0 | 38,7 - 78,9 | 69,0 | 49,2 - 84,7 | 1,93 | 0,34 - 0,99 | 0,58 | 0,58 - 0,99 |  |
| ≤32 | 68,0 | 46,5 - 85,1 | 69,0 | 49,2 - 84,7 | 2,19 | 0,25 - 0,86 | 0,46 | 0,46 - 0,86 |  |
| ≤33 | 72,0 | 50,6 - 87,9 | 65,5 | 45,7 - 82,1 | 2,09 | 0,22 - 0,85 | 0,43 | 0,43 - 0,85 |  |
| ≤34 | 80,0 | 59,3 - 93,2 | 55,2 | 35,7 - 73,6 | 1,78 | 0,15 - 0,85 | 0,36 | 0,36 - 0,85 |  |
| ≤35 | 80,0 | 59,3 - 93,2 | 51,7 | 32,5 - 70,6 | 1,66 | 0,16 - 0,91 | 0,39 | 0,39 - 0,91 |  |
| ≤36 | 80,0 | 59,3 - 93,2 | 44,8 | 26,4 - 64,3 | 1,45 | 0,18 - 1,08 | 0,45 | 0,45 - 1,08 |  |
| ≤38 | 84,0 | 63,9 - 95,5 | 44,8 | 26,4 - 64,3 | 1,52 | 0,13 - 0,96 | 0,36 | 0,36 - 0,96 |  |
| ≤39 | 88,0 | 68,8 - 97,5 | 41,4 | 23,5 - 61,1 | 1,50 | 0,092 - 0,91 | 0,29 | 0,29 - 0,91 |  |
| ≤40 | 88,0 | 68,8 - 97,5 | 34,5 | 17,9 - 54,3 | 1,34 | 0,11 - 1,13 | 0,35 | 0,35 - 1,13 |  |
| ≤42 | 88,0 | 68,8 - 97,5 | 17,2 | 5,8 - 35,8 | 1,06 | 0,18 - 2,63 | 0,7 | 0,70 - 2,63 |  |
| ≤44 | 88,0 | 68,8 - 97,5 | 13,8 | 3,9 - 31,7 | 1,02 | 0,21 - 3,52 | 0,87 | 0,87 - 3,52 |  |
| ≤45 | 88,0 | 68,8 - 97,5 | 10,3 | 2,2 - 27,4 | 0,98 | 0,26 - 5,24 | 1,16 | 1,16 - 5,24 |  |
| ≤46 | 92,0 | 74,0 - 99,0 | 6,9 | 0,8 - 22,8 | 0,99 | 0,18 - 7,64 | 1,16 | 1,16 - 7,64 |  |
| ≤48 | 96,0 | 79,6 - 99,9 | 3,5 | 0,09 - 17,8 | 0,99 | 0,076 - 17,60 | 1,16 | 1,16 - 17,60 |  |
|  |  |  |  |  |  |  |  |  |  |
| **Cutoff points** | | **Sensitivity (%)** | **95% CI Sensitivity** | **Specificity (%)** | **95% CI Specificity** | **LR+** | **95% CI LR+** | **LR-** | **95% CI LR-** |
| **Women** |  |  |  |  |  |  |  |  |  |
| ≤10 | 12,0 | 2,5 - 31,2 | 96,2 | 80,4 - 99,9 | 3,12 | 0,35 - 28,03 | 0,92 | 0,72 - 1,05 |  |
| ≤11 | 16,0 | 4,5 - 36,1 | 96,2 | 80,4 - 99,9 | 4,16 | 0,50 - 34,71 | 0,87 | 0,69 - 1,09 |  |
| ≤12 | 20,0 | 6,8 - 40,7 | 92,3 | 74,9 - 99,1 | 2,60 | 0,55 - 12,19 | 0,87 | 0,69 - 1,09 |  |
| ≤14 | 28,0 | 12,1 - 49,4 | 88,5 | 69,8 - 97,6 | 2,43 | 0,70 - 8,35 | 0,81 | 0,61 - 1,08 |  |
| ≤16 | 28,0 | 12,1 - 49,4 | 84,6 | 65,1 - 95,6 | 1,82 | 0,61 - 5,46 | 0,85 | 0,63 - 1,14 |  |
| ≤17 | 32,0 | 14,9 - 53,5 | 84,6 | 65,1 - 95,6 | 2,08 | 0,72 - 6,05 | 0,8 | 0,59 - 1,10 |  |
| ≤18 | 36,0 | 18,0 - 57,5 | 76,9 | 56,4 - 91,0 | 1,56 | 0,65 - 3,74 | 0,83 | 0,58 - 1,19 |  |
| ≤18,5 | 40,0 | 21,1 - 61,3 | 76,9 | 56,4 - 91,0 | 1,73 | 0,74 - 4,06 | 0,78 | 0,53 - 1,14 |  |
| ≤20 | 56,0 | 34,9 - 75,6 | 73,1 | 52,2 - 88,4 | 2,08 | 1,01 - 4,28 | 0,6 | 0,37 - 0,99 |  |
| ≤21 | 60,0 | 38,7 - 78,9 | 69,2 | 48,2 - 85,7 | 1,95 | 1,01 - 3,77 | 0,58 | 0,34 - 1,00 |  |
| ≤22 | 60,0 | 38,7 - 78,9 | 61,5 | 40,6 - 79,8 | 1,56 | 0,87 - 2,79 | 0,65 | 0,37 - 1,15 |  |
| ≤23 | 68,0 | 46,5 - 85,1 | 57,7 | 36,9 - 76,6 | 1,61 | 0,95 - 2,71 | 0,55 | 0,29 - 1,07 |  |
| ≤24 | 80,0 | 59,3 - 93,2 | 57,7 | 36,9 - 76,6 | 1,89 | 1,16 - 3,09 | 0,35 | 0,15 - 0,81 |  |
| ≤26 | 88,0 | 68,8 - 97,5 | 53,9 | 33,4 - 73,4 | 1,91 | 1,23 - 2,96 | 0,22 | 0,073 - 0,68 |  |
| ≤28 | 88,0 | 68,8 - 97,5 | 46,2 | 26,6 - 66,6 | 1,63 | 1,11 - 2,40 | 0,26 | 0,083 - 0,81 |  |
| ≤29 | 88,0 | 68,8 - 97,5 | 38,5 | 20,2 - 59,4 | 1,43 | 1,02 - 2,00 | 0,31 | 0,097 - 1,00 |  |
| ≤30 | 96,0 | 79,6 - 99,9 | 30,8 | 14,3 - 51,8 | 1,39 | 1,06 - 1,81 | 0,13 | 0,018 - 0,97 |  |
| ≤31 | 96,0 | 79,6 - 99,9 | 26,9 | 11,6 - 47,8 | 1,31 | 1,03 - 1,68 | 0,15 | 0,020 - 1,12 |  |
| ≤32 | 96,0 | 79,6 - 99,9 | 19,2 | 6,6 - 39,4 | 1,19 | 0,97 - 1,46 | 0,21 | 0,026 - 1,66 |  |
| ≤33 | 96,0 | 79,6 - 99,9 | 15,4 | 4,4 - 34,9 | 1,13 | 0,95 - 1,36 | 0,26 | 0,031 - 2,17 |  |
| ≤34 | 96,0 | 79,6 - 99,9 | 3,9 | 0,10 - 19,6 | 1,00 | 0,89 - 1,12 | 1,04 | 0,069 - 15,74 |  |

**Note:** CI: confidence interval; LR+: positive Log-likelihood; LR−: negative Log-likelihood*.*
